# Skin Manifestations Associated With Mantle Cell Lymphoma: a Case Report

**DOI:** 10.4084/MJHID.2013.020

**Published:** 2013-02-25

**Authors:** Avinash Kumar Singh, Gaurav Dixit, Sanjeev Sharma, Suman Kumar, Rajni Yadav, Narendra Agrawal, Pravas Mishra, Tulika Seth, C. Sarkar, M. Mahapatra, Renu Saxena

**Affiliations:** 1 Department of Hematology, All India Institute of Medical Sciences, New Delhi; 2 Department of Pathology, All India Institute of Medical Sciences, New Delhi

## Abstract

Mantle cell lymphoma (MCL) is a distinct non-Hodgkin’s lymphoma type that commonly affects extra nodal sites. The most often affected sites are bone marrow, gastrointestinal tract and Waldeyer’s ring, being the skin rarely involved. We report a case of 56 year-old man with MCL, exhibiting multiple large maculopapular skin rashes and skin ulcers. Histopathological examination had not shown direct infiltration by any atypical cells. He had significant improvement of skin lesions with combination chemotherapy and debridement. Awareness of skin manifestations of MCL is crucial for dermatologists and haematologists to establish the early diagnosis and timely administration of appropriate treatment.

## Case Report

**Figure 1:**
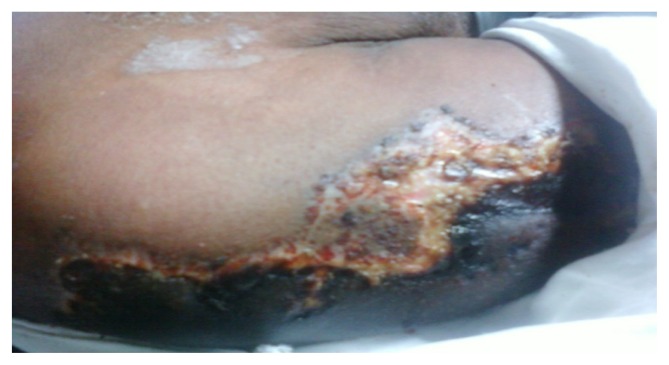
Skin lesion with central necrosis over right arm

**Figure 3:**
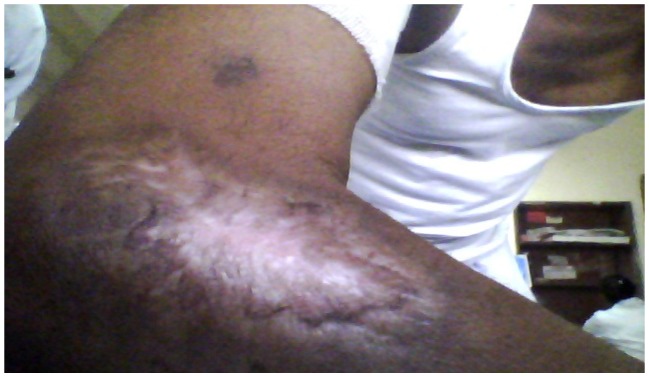
Skin lesion after 4 months

A 56 years old indo-aryan black gentleman, farmer by occupation, occasional smoker and alcoholic for more than 20 years, presented to Haematology OPD with complains of weakness and easy fatigability for one month, he also had glandular swellings involving the neck, axilla and inguinal region. He noticed erythematous maculopapular rashes over both upper limbs, 2 days back. There was no history of fever, cough, bleeding, skeletal pain or jaundice. There was no involvement of the oral mucosa by the skin lesions. The past and family history was not significant. On examination, he had generalised lymphadenopathy and massive hepatosplenomegaly. The skin rashes (Figure 1) were maculopapular erythematous, size varying from 4×5 cm to 10×8 cm without any initial itching, pain or secretion. Over 4 to 5 days, the rashes developed blackish discolouration, followed by bullae formation that eventually ruptured. The blood exam revealed anemia, thrombocytopenia and leucocytosis (WBC = 18000/mm^3^) with presence of atypical lymphoid cells on peripheral smear. The bone marrow examination showed 45% immature lymphoid cells, negative for MPO, SB, AP, NSE & PAS on cytochemistry. The bone marrow biopsy showed infiltration by atypical lymphoid cells. Immunophenotyping by flow cytometry of bone marrow showed positivity for co-expression of CD19/5, CD19, FMC7, Kappa clonality, CD25, HLADR, CD22, CD20, CD79b, SIgM and negative for co-expression of CD23/5, CD10, CD23, CD11C, CD103, CD2, CD3, CD34, SIgG, TdT. The axillary lymph node biopsy was suggestive of blastoid variant of mantle cell lymphoma, which showed immunopositivity for CD 20, CD43, Cyclin D1, BCL-2, BCL-6 and immunonegative for CD5, CD3, CD10, CD23. The skin biopsy from the left arm maculopapular lesion (including both healthy and affected area) showed congested vessels along with extravasations of RBC in the dermis without any atypical lymphoid cells (Figure 2a and 2b). He received R-CHOP chemotherapy and has undergone debridement (Figure 3) for the skin lesions. Gradually, after 5 cycles of chemotherapy, he has attained complete remission of skin lesions along with disappearance of lymphadenopathy, organomegaly and normalisation of the hemogram.

## Discussion

Mantle cell lymphoma (MCL) is a malignant tumor derived from B cells in the mantle zone of lymphoid follicles characterized by specific pathologic, immunophenotypic and molecular genetics features, and usually takes an aggressive clinical course, defined in the World Health Organization (WHO) classification.^1^–^3^ Histologically, it shows a diffuse or nodular monotonous proliferation of small lymphoid cells with scant cytoplasm and irregular nuclear contours in lymphnodes.

Immunophenotypically, the tumor cells are positive for B-cell markers CD79a, CD19, CD20 and CD22 as well as CD5, and are usually negative for CD10 and CD23.^5^,^6^ The majority of MCL have an associated cytogenetic abnormality t(11,14) (q13;q32) translocation, which causes a juxtaposition of the *CCND-1* gene on chromosome 11 with the immunoglobulin heavy chain gene on chromosome 14, resulting in an over expression of cyclin D1 protein, that leads to a positive signal for transition to the S phase.^7^–^9^ Cyclin D1 over expression is considered to be an important diagnostic marker for MCL.^10^

MCL frequently involves extra nodal organs, particularly the bone marrow, gastrointestinal tract and Waldeyer’s ring; thus it has been considered in the past as an extracutaneous lymphoma. However, MCL rarely affects the skin. In two large series of 121 and 59 cases of MCL, only 3 and 2 patients respectively had skin lesions, respectively.^3^,^4^ To the best of our knowledge, only 18 cases of mantle cell lymphoma with skin manifestation have been reported in English literature (Table 1).^11^,^12^ The commonly reported sites of skin involvement in MCL are trunk (60%), face (30%) followed by arm (20%), thigh, leg & scalp.^11^,^12^ Skin lesions manifested as nodular lesions in 6 patients (34%), macular or maculopapular lesions, as in our case, in 6 (34%), tumoral or infiltrated plaques in 4 (21%), and subcutaneous nodules in 2 (11%).^11^,^12^

In most of the reported cases of skin manifestations of MCL, skin lesion has shown infiltration by atypical lymphoid cells, while a reactive cutaneous eruption, simulating insect bites, has been only incidentally described in association with MCL.^11^,^13^–^15^ The skin involvement, even if rare, has been most frequently reported in the blastoid variant of MCL as in our case; when present the skin localizations are disseminated and associated with a poor prognosis.^11^,^12^,^16^ Although, large reactive skin lesions have not been reported previously, the absence of atypical lymphoid cells and the complete remission with chemotherapy suggest that the skin lesions should be considered in the present case as reactive to mantle cell lymphoma; however, the presence of hidden malignant cells in the lesion cannot be excluded. The prognosis of MCL with skin localizations is particularly poor; aggressive chemotherapy may improve the survival rate.^12^ As cutaneous lesions can be the first manifestation of MCL, awareness of MCL is crucial for dermatologists and haematologists to establish an early diagnosis and perform an appropriate treatment.

## Figures and Tables

**Figure 2a:**
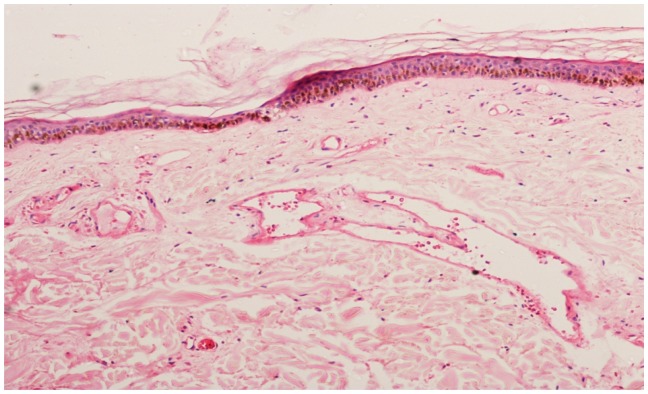
Skin biopsy showing dilated and congested vessels

**Figure 2b:**
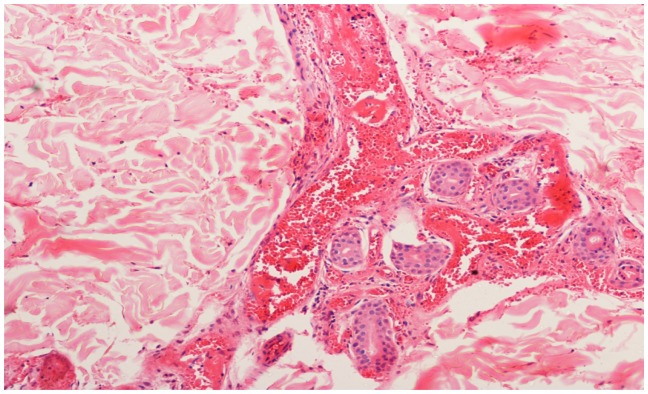
Skin biopsy showing extravasation RBC in the dermis

**Table 1:** 

No	Author	Age/Sex	Location	Clinical presentation	Extracutaneous Involvement	Stage	Prognosis
1	Ellison	66M	Temple	Macular skin lesions	LN, liver, spleen, lung, pleural cavity, CNS	IV	D (55d; after initial hospitalization)
2	Geerts	65F	Forehead	Nodules	LN, BM	IVA	D (1.5y; after diagnosis)
3	Geerts	77F	Back, breast, arm	Tumoral plaques	Bronchus wall	IVA	
4	Bertero	51M	Breast	Subcutaneous nodule	LN, liver, spleen	IVA	A (17y; after onset)
5	Bertero	78F	Breast, back	Nodules	None	I E	D (3y; after diagnosis)
6	Bertero	43M	Back, face, arm	Infiltrated plaques	LN, liver, spleen	IVA	A
7	Bertero	22M	Breast	Nodules	None	I E	A
8	Marti	61F	Abdomen	Tumoral plaque	LN, BM, tonsils	IVA	D (15m; after diagnosis)
9	Moody	47M	Ear	Nodules	LN, BM	IVA	A (3y; after onset)
10	Dubus	56M	Breast, back	Erythematous papules	LN, BM, PB	IVA	D (1y; after treatment)
11	Dubus	89M	Breast, back, abdomen	Infiltrated purpuric papules	LN, BM, PB	IVA	D (5d; after diagnosis)
12	Dubus	72M	Face, breast, arm, axilla	Subcutaneous nodules	LN, BM	IVA	A (1y; after treatment)
13	Sen	85M	Leg	Macular rash	LN. BM, buccal mucosa	IVB	D (20m; after onset)
14	Sen	76M	Thigh	Nodule	None	I E	A (30m; after onset)
15	Sen	56M	Chest	Nodules	BM, GI	IVA	A (21m; after onset)
16	Sen	57M	Legs	Maculopapular rash	LN, BM, PB	IVB	D (9m; after onset)
17	Sen	61M	Flank back, thigh	Plaques	LN, BM, PB leptomeninges	IVB	D (15m; after onset)
18	Motegi	62 M	Back, upper extremities	nodules	LN, Spleen, gastric mucosa, tonsils	IVA	A (4 month, after treatment)
